# Pulmonary Artery Aneurysm (PAA) in Behçet's Disease Presenting With Recurrent Hemoptysis: A Case Report

**DOI:** 10.1002/ccr3.70663

**Published:** 2025-07-27

**Authors:** Shahabaldin Sorouri, Maryam Safari Farmad, Amirhossein Amiriani, Amir Baniasad

**Affiliations:** ^1^ Lung Disease Research Center Mashhad University of Medical Sciences Mashhad Iran; ^2^ Student Research Committee Faculty of Medicine, Mashhad University of Medical Sciences Mashhad Iran

**Keywords:** autoimmune vasculitis, Behçet's disease, CT pulmonary angiogram, peripheral pulmonary artery aneurysm, recurrent hemoptysis

## Abstract

Pulmonary artery aneurysm is a serious but under‐recognized complication of Behçet's disease that may present with hemoptysis. Early diagnosis coupled with timely initiation of immunosuppressive therapy is crucial in preventing aneurysmal progression and associated life‐threatening complications.

## 1 Introduction

Behçet's disease (BD) is a chronic, multisystem inflammatory disorder characterized by recurrent episodes of acute inflammation affecting blood vessels of all sizes and types, with venous involvement more common than arterial. The disease typically begins in the third decade of life and tends to present with more severe symptoms in males than in females [1].

The classic triad of oral and genital ulcers and recurrent uveitis is characteristic of BD. In addition, various clinical symptoms may manifest in other areas, including the skin, joints, gastrointestinal tract, genitourinary tract, central nervous system, cardiovascular system, and lungs [2].

Pulmonary complications are observed in approximately 5% of BD patients, with a reported prevalence ranging from 1% to 7.7% [3, 4]. Pulmonary artery involvement usually develops 3–4 years after disease onset and may include aneurysms, thrombosis, parenchymal abnormalities, and infarctions. Among these, pulmonary artery aneurysm (PAA) is the most frequent and potentially life‐threatening manifestation [4, 5].

PAAs are the second most common arterial involvement in BD and may result in severe bleeding if they rupture into the bronchial system. Therefore, early diagnosis and treatment of PAA in BD are crucial [6].

Computed tomography (CT) is the most suitable noninvasive diagnostic method as it provides excellent vascular images with a small amount of contrast agent over a short period. It is valuable both for initial diagnosis and follow‐up of PAA in BD [3].

This case report presents a rare case of BD manifesting as recurrent hemoptysis due to PAA. It highlights the importance of a thorough diagnostic evaluation and timely intervention to prevent potentially fatal complications.

## 2 Case History/Examination

A 31‐year‐old male was referred to our tertiary hospital with a chief complaint of recurrent non‐massive hemoptysis (1–2 cc, occurring 1–2 times per month) over the past 7 months. He reported dyspnea during mild exertion (e.g., walking up a slight hill or climbing 10 stairs) and an occasional nonproductive cough. Two years earlier, he had experienced urogenital lesions and recurrent oral aphthae. Seven months ago, following an episode of hemoptysis initially attributed to pneumonia, he was prescribed prednisolone and a short course of oral antibiotics. The oral and urogenital lesions diminished with prednisolone (7.5 mg daily), which the patient continued at the initial dose.

**FIGURE 1:**
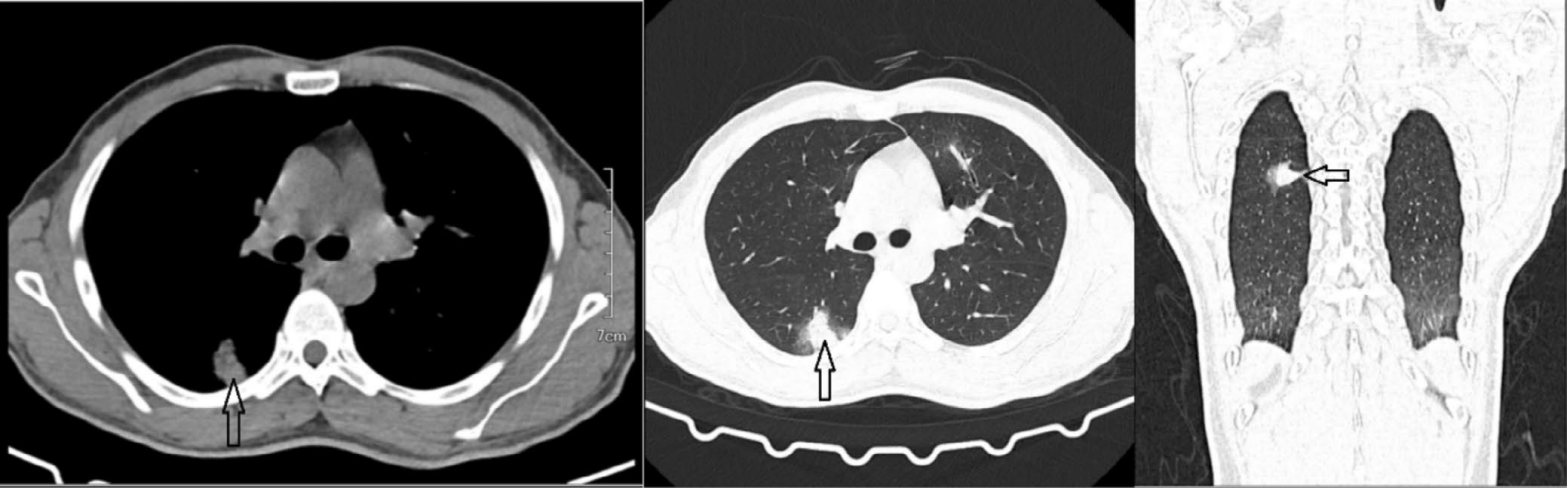
The chest CT scan revealed an 8‐mm nodule in the RLL (highlighted by the arrows).

Considering recurrent hemoptysis, the patient was referred to a pulmonologist 6 months ago, and a chest CT scan was performed (Figure 1), which revealed an 8‐mm nodule in the right lower lobe (RLL). A subsequent bronchoscopy revealed gross bleeding in the RLL bronchus, but Ziehl–Neelsen (ZN) and real‐time polymerase chain reaction assays of the bronchial fluid were negative for the *Mycobacterium tuberculosis* complex. Additionally, bronchial fluid cytology showed no evidence of malignancy.

Considering these negative results, a transthoracic needle biopsy was performed, yielding findings compatible with alveolar hemorrhage and organizing pneumonia. As a result, treatment with prednisolone was continued. Currently, the patient is admitted to our hospital with the chief complaint of recurrent non‐massive hemoptysis. He did not report any arthralgia, morning stiffness, or a history of smoking or opium addiction. His vital signs included a blood pressure of 110/70 mmHg, a pulse rate of 85 beats per minute, a respiratory rate of 14 breaths per minute, and a temperature of 37.5°C. Lung auscultation was clear bilaterally, and no oral or urogenital lesions were found upon physical examination. Additionally, no pathological findings were observed on joint examination, and no skin lesions were noted.

## 3 Differential Diagnosis, Investigations, and Treatment

**FIGURE 2:**
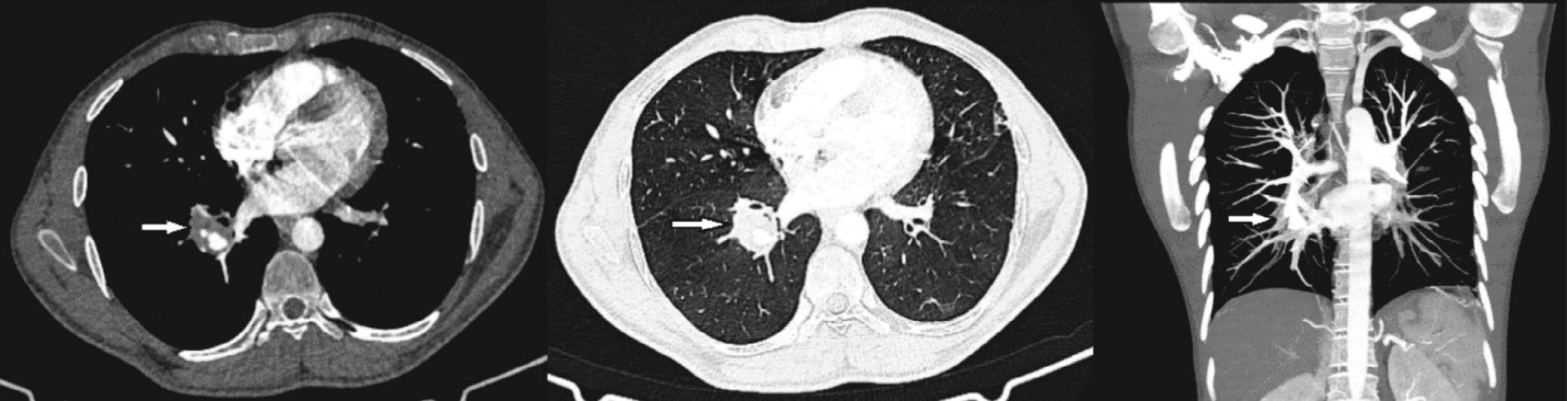
CTPA revealed aneurysmal dilatation of the right interlobar descending artery containing a clot with an outer diameter of 19 mm (highlighted by the arrows).

Considering recurrent hemoptysis, a CT pulmonary angiogram (CTPA) was performed (Figure 2). In CTPA, aneurysmal dilation of the right interlobar descending artery containing a clot with an external diameter of 19 mm was observed. The central canal was open and extended peripherally. No abnormal intraluminal filling defect suggesting pulmonary embolism was observed in other pulmonary arterial structures. A rheumatological consultation and laboratory tests were performed; the results are shown in Table 1.

**TABLE 1:** Laboratory findings of the patient.

Test name	Result	Reference range
Fluorescent antinuclear Ab (titer)	Negative < 1/80	< 1/80
Anti‐dsDNA (IU/mL)	2.1	< 100
C3 (mg/dL)	71	55–120
C4 (mg/dL)	22	10–40
ANA profile		
dsDNA	Negative	
Nucleosome	Negative	
Sm	Negative	
P0	Negative	
Histone	Negative	
CENP B	Negative	
U1‐snRNP	Negative	
SSA/Ro60	Negative	
SSA/Ro52	Negative	
SSB/La	Negative	
Scl/70	Negative	
Jo‐1	Negative	
Anti‐cardiolipine Ab (IgG) (GPL/mL)	0.8	< 10
Anti‐cardiolipine Ab (IgM) (MPL/mL)	2.0	< 10
Beta‐2 glycoprotein 1 Ab (IgG) (U/mL)	2.1	< 10
Beta‐2 glycoprotein 1 Ab (IgM) (U/mL)	2.4	< 10
HLA B5	Positive	
Lupus anticoagulant	Negative	

Abbreviations: Ab, antibody; ANA, antinuclear antibody; anti‐dsDNA, anti‐double‐stranded DNA; CENP‐B, anti‐centromere protein‐B; HLA, human leukocyte antigen; Ig, immunoglobulin; Jo‐1, anti‐histidyl‐tRNA synthetase; Scl/70, antitopoisomerase I; Sm, Smith; SSA/Ro, Sjogren's syndrome‐related antigen A; SSB/La, Sjogren's‐syndrome‐related antigen B; U1‐snRNP, U1 small nuclear ribonucleoprotein particle.

In echocardiography, the patient had an ejection fraction of 55% without abnormal findings. Abdominal and brain CT angiography showed no evidence of aneurysms or vascular malformation. Ophthalmologic evaluation using a slit lamp also revealed no signs of uveitis and retinal vasculitis, although our examination did not include posterior segment evaluation.

## 4 Outcome and Follow‐Up

Regarding the positive result of HLA B5, previous oral and urogenital lesions, and aneurysm of the right interlobar descending artery, the diagnosis of BD was confirmed for the patient. Treatment with methylprednisolone (1 g daily for 3 days) was initiated, followed by colchicine (1 mg daily) and prednisolone (50 mg daily). The prednisolone dose was tapered after 10 days until the maintenance dose of 7.5 mg daily was reached. Anticoagulant therapy was not administered to the patient as the risks of bleeding, particularly the potential for exacerbating aneurysm rupture, and the treatment strategy focused on controlling the underlying vasculitis and inflammatory process.

The patient was scheduled for close follow‐up, including clinical evaluations every 3 months during the first year, with a focus on respiratory symptoms, mucocutaneous recurrence, and visual changes. Laboratory tests, including ESR and CRP, will be monitored at each visit to assess systemic inflammation. Repeat CT pulmonary angiography is planned at 3 and 6 months to monitor aneurysm morphology. In long‐term follow‐up, annual imaging may be considered if stability is confirmed.

## 5 Discussion

BD is a chronic systemic inflammatory disorder that primarily affects individuals between the ages of 20 and 40. While the disease affects both men and women, there is a notable difference in gender distribution, with men being more commonly affected in Middle Eastern countries. In contrast, women tend to be more frequently diagnosed in the United States and the United Kingdom [2]. In this case, the patient was a 31‐year‐old male.

As it is a multisystemic disease, clinical manifestations can affect almost the entire body. The most common manifestations include ocular involvement, as well as genital and oral aphthosis. Other symptoms, such as skin lesions, neurological and vascular manifestations, joint involvement, gastrointestinal manifestations, epididymitis, and pleuropulmonary and cardiac manifestations, have also been reported [7, 8]. In our patient, the initial symptoms included recurrent urogenital lesions and aphthae over the past 2 years, followed by recurrent hemoptysis and dyspnea over the past 7 months.

Pulmonary complications, particularly hemoptysis, are among the most common manifestations of BD. Hemoptysis is usually associated with PAAs, which can be life‐threatening due to the risk of rupture. The pathophysiology of hemoptysis in BD is linked to aneurysms eroding into the bronchial system as well as in situ thrombosis due to active vasculitis [8, 9].

Pulmonary involvement in BD is not limited to the pulmonary artery. BD can also cause other types of pulmonary diseases, such as nodules, cavities, atelectasis, hemorrhages, and infarcts in the lung parenchyma, which may be mistaken for infection but are often associated with pulmonary artery involvement, especially thrombosis of the pulmonary vessels [5, 10, 11].

PAA is the most common pulmonary manifestation and a known cause of mortality and morbidity in BD [4].

PAA formation in BD results from a triad of neutrophilic vasculitis, vasa vasorum inflammation, and thrombotic complications. Activated neutrophils infiltrate the arterial wall, releasing proteases (e.g., Matrix Metalloproteinases (MMPs), elastase) that degrade structural proteins. Meanwhile, cytokines (IL‐1α and tumor necrosis factor [TNF]‐β) amplify vascular inflammation, especially in the vasa vasorum [12]. BD's prothrombotic state—driven by endothelial dysfunction, autoantibodies, and impaired fibrinolysis—promotes mural thrombosis, creating a vicious cycle of thrombosis and inflammation that culminates in aneurysmal dilation [13].

BD‐related PAAs are typically found in the right lower lobe arteries (RLL), followed by the right and left main pulmonary arteries [14]. In our patient, an initial CT scan revealed an 8‐mm nodule in the RLL, which was later found to be associated with aneurysmal dilatation of the right interlobar descending artery.

The gold standard for diagnosing PAAs in BD is a pulmonary angiogram. However, due to the high risk of stimulation and exacerbation of pulmonary artery thrombosis by the mechanism of venous puncture or rapid injection of contrast agent, spiral computed tomography angiogram (CTPA) is often used as an alternative diagnostic tool [14, 15].

Additionally, in patients for whom CTPA is contraindicated, such as those with renal failure or severe iodine allergy, magnetic resonance imaging can be used as an alternative [16]. Other diagnostic modalities, such as FDG‐PET, can help identify active vasculitis, and echocardiography can evaluate potential hemodynamic consequences, including pulmonary hypertension [17]. Additionally, laboratory tests should include thrombophilia screening (e.g., antiphospholipid antibodies) to assess recurrent thrombosis, though acute‐phase reactants may be influenced by systemic inflammation [18].

If left untreated, PAAs in BD may lead to severe complications, including aneurysm rupture, pulmonary embolism, and pulmonary hypertension, all of which can cause respiratory failure and increase mortality risk. Immunosuppressive therapy is crucial in managing these patients, as it helps control systemic inflammation, reduce the prothrombotic state, and stabilize the aneurysms. By managing the underlying vasculitis, immunosuppression improves long‐term outcomes and minimizes the risk of life‐threatening events [16, 19].

Immunosuppressive drugs, such as corticosteroids and cyclophosphamide, are the preferred first‐line immunosuppressive therapy for BD‐related PAAs. In many cases, immunosuppressive therapy alone may resolve the aneurysms, but endovascular or surgical intervention may be necessary for patients who experience massive hemoptysis, rapidly enlarging aneurysms, or rupture [19, 20, 21].

Open surgery and endovascular procedures, including embolization with acrylic glue, coils, plugs, occluders, or stents, can also be used for PAA in BD [14, 15, 16]. However, these interventions carry risks, particularly in active vasculitis, such as pseudoaneurysm formation, procedure‐related bleeding, and high perioperative morbidity and mortality. Endovascular procedures tend to have fewer risks compared to open surgery but still present challenges, such as contrast‐induced nephropathy, arterial dissection, arterial thrombosis, nontarget embolization, and lung infarction [16].

Although other vasculitides, such as Takayasu arteritis (TAK) and granulomatosis with polyangiitis (GPA), can present with PAAs, the pathophysiology and clinical management differ significantly in BD. BD is a neutrophilic, non‐granulomatous vasculitis that affects both arteries and veins and often leads to bilateral, rupture‐prone PAAs. In contrast, TAK is characterized by granulomatous inflammation and stenosis, and GPA presents with necrotizing granulomas and cavitary lung nodules [22, 23, 24]. BD requires aggressive immunosuppression to control inflammation, typically with antitumor necrosis factor (TNF) agents. Anticoagulation is generally avoided in BD due to the increased risk of aneurysm rupture [25]. In contrast, TAK responds to IL‐6 inhibitors (e.g., tocilizumab), and GPA can be managed with rituximab or cyclophosphamide [26, 27].

The prognosis depends mainly on the size of the aneurysm; those larger than 3 cm carry a higher risk of mortality [11]. Prompt recognition, appropriate immunosuppressive therapy, and careful monitoring are crucial for improving outcomes in these patients [11].

## 6 Conclusion

This case highlights the critical importance of considering BD in patients with unexplained hemoptysis, particularly when accompanied by a history of mucocutaneous lesions. Though rare, PAA is a potentially fatal complication of BD that requires high clinical suspicion and prompt imaging for diagnosis. Our report emphasizes the importance of striking a balance between immunosuppressive therapy and the avoidance of anticoagulation in managing BD‐related PAA, aiming to control inflammation while minimizing the risk of rupture. While endovascular or surgical options exist, their risks must be carefully weighed in the context of active vasculitis. Early diagnosis, tailored treatment, and structured follow‐up are key to improving outcomes in these complex, high‐risk patients.

## Author Contributions

**Shahabaldin Sorouri:** conceptualization, investigation, supervision, writing – original draft, writing – review and editing. **Maryam Safari Farmad:** data curation, investigation, writing – original draft, writing – review and editing. **Amirhossein Amiriani:** data curation, investigation, writing – original draft, writing – review and editing. **Amir Baniasad:** conceptualization, data curation, investigation, supervision, writing – original draft, writing – review and editing.

## Consent

Written informed consent was obtained from the patient to publish this report in accordance with the journal's patient consent policy.

## Conflicts of Interest

The authors declare no conflicts of interest.

## Data Availability

The data supporting this study's findings are available from the corresponding author upon reasonable request.
